# P16/Ki67 Dual Staining in Glandular Cell Abnormalities of the Uterine Cervix

**DOI:** 10.3390/cancers16091621

**Published:** 2024-04-23

**Authors:** Saša Jeromel, Alenka Repše Fokter, Andraž Dovnik

**Affiliations:** 1 Medical Faculty, University of Maribor, Taborska ulica 8, 2000 Maribor, Slovenia; sasa.skrbinsek@student.um.si; 2 Department of Pathology and Cytology, General Hospital Celje, Oblakova 5, 3000 Celje, Slovenia; 3 University Clinic for Gynaecology and Obstetrics, University Medical Centre Maribor, Ljubljanska 5, 2000 Maribor, Slovenia

**Keywords:** p16/Ki67 dual staining, glandular cell abnormalities, adenocarcinoma in situ, cervical adenocarcinoma

## Abstract

**Simple Summary:**

The triage of glandular cell abnormalities of the uterine cervix poses many challenges due to high interobserver variability. p16/Ki67 dual staining has been proposed as an objective test that could decrease interobserver variability in diagnosing atypical glandular cells. Our results indicate the possible role of p17/Ki67 dual staining in triaging glandular cell abnormalities.

**Abstract:**

Very limited information exists about the role of p16/Ki67 dual staining on glandular cells in detecting glandular precancerous lesions and cervical adenocarcinoma. In this study, we investigated the diagnostic accuracy of p16/Ki67 dual staining for the detection of glandular and squamous lesions on the uterine cervix and for cancer of the upper reproductive tract. We performed a retrospective analysis of prospectively collected data on 96 patients with glandular cell abnormalities. We analyzed the diagnostic accuracy of p16/Ki67 dual staining for atypical glandular cells, not otherwise specified (AGC-NOS); atypical glandular cells, favor neoplastic (AGC-FN); adenocarcinoma in situ (AIS); and A-CA (cervical adenocarcinoma). A separate analysis for the detection of squamous precancerous lesions and squamous-cell carcinoma (CIN3+) and for cancer of the upper reproductive tract (EC/OC) was performed. Among patients who had normal histology or a low-grade lesion on final analysis, only 8.5% had positive dual staining. On the other hand, 85.7% of patients with AIS+ on final histology had positive dual staining. The respective specificities of p16/Ki67 dual staining on AGC-NOS for the detection of AIS+ (adenocarcinoma in situ or cervical adenocarcinoma), CIN3+ and EC/OC were 91.5%, 88.7% and 86.4%. High specificity values of p16/Ki67 dual staining on cervical smears labelled as AGC-NOS for the detection of CIN3+ and AIS+ suggest that this method might be a useful addition in cervical cancer screening.

## 1. Introduction

The examination of cells from the surface of the uterine cervix, either by conventional or liquid-based cytology, is one of the main methods for cervical cancer screening and has been in use for many decades [1]. Since the majority of cervical cancers develop as a result of persistent infection with high-risk human papillomaviruses (hr-HPV), primary HPV screening has become a part of screening protocols in many countries [2,3]. Both methods have certain limitations [4]. HPV screening is less useful in women younger than 30 years old because the majority of HPV infections are productive and do not cause cervical dysplasia, which leads to lower test specificity [4]. On the other hand, cytology is associated with a high rate of false negative results, which makes frequent repetitions of this test imperative to increase its efficiency [5,6]. The next step in the process of diagnosis of cervical precancerous lesions is a referral to colposcopy [4]. However, the referral of all HPV-positive women to colposcopy would increase the costs of screening programs. Therefore, an efficient triage method is needed [4]. Among these, HPV genotyping, HPV methylation and p16/Ki67 dual staining (DS) have been most extensively investigated [7].

The role of p16/Ki67 DS in cervical cancer screening has been widely researched and compared to conventional cytology and HPV testing [8,9,10,11]. In case of persistent infection with hr-HPV, the DNA of the virus integrates into the host DNA, and this in turn causes an interaction between early HPV genes E6 and E7 and tumor suppressor genes p53 and Rb [12]. The result of this interaction is uncontrolled cellular division [12]. The dysfunctional Rb causes an accumulation of the tumor suppressor protein p16, which aims to slow down the progression of the cell cycle [13]. The accumulation of p16 in the nucleus and in the cytoplasm of HPV-infected cells can be detected by immunostaining [14]. On the other hand, Ki67 is a proliferation marker, and the concurrent expression of p16 and Ki67 indicates oncologic transformation of an hr-HPV-infected cell [15]. p16/Ki67 DS has shown higher sensitivity for the detection of cervical intraepithelial neoplasia grade 2 or higher (CIN2+) compared to conventional cytology, in addition to comparable specificity [8,11]. Compared to HPV testing, p16/Ki67 DS has shown similar sensitivity and higher specificity for the detection of CIN2+ [10,15,16].

However, the majority of the studies performed in the past focused on the utility of p16/Ki67 DS on squamocellular cytologic changes. Only very limited information exists about the usefulness of p16/KI67 in the detection of glandular cervical precancerous lesions and cervical adenocarcinoma [17]. The detection of glandular precancerous lesions by cervical cytology or by colposcopy is often prevented by the presence of a glandular lesion at the base of the crypts and by the often-multifocal nature of adenocarcinoma in situ (AIS) [10,17]. Therefore, a method to increase the reliability of cervical cytology in detecting glandular lesions would be very helpful, especially in light of the increasing incidence of cervical adenocarcinoma [18]. In this study, we investigated the utility of p16/Ki67 DS on atypical glandular cells for the detection of glandular and squamocellular changes and for the detection of cancer of the upper reproductive tract.

## 2. Materials and Methods

### 2.1. Study Design and Patient Selection

We performed a retrospective analysis of prospectively collected data. This study was approved by the institutional ethics committee (approval No. 53/2022/2). Among 167,692 cervical smears investigated at our institution in the period 2018–2021, glandular changes were found in 121 cases. Within this group, 96 samples were subjected to p16/Ki67 DS. The glandular changes were classified according to the Bethesda classification as AGC-NOS (atypical glandular cells, not otherwise specified), AGC-FN (atypical glandular cells, favor neoplastic), AIS (adenocarcinoma in situ) and A-CA (adenocarcinoma). In total, 96 patients with glandular cell abnormalities and DS results had available histological reports from surgical specimens. 

We excluded the smears with endometrial cells and those with concurrent squamous and glandular cytologic changes. In addition, we excluded cases of glandular changes without performed DS and cases where only biopsy from a colposcopy was available. Follow-up data with histological reports of surgical specimens were available for all included patients. According to the national guidelines, all patients with glandular cytological changes undergo colposcopy, endocervical curettage and transvaginal ultrasound examination. If endocervical curettage is negative for high-risk lesions, conization is performed. In case of suspicious findings on ultrasound examination, patients are referred either for hysteroscopy or for additional preoperative imaging. Histological samples from the follow-up obtained either by large loop excision of the transformation zone or by hysterectomy are analyzed. This analysis includes patients who had hysterectomy performed.

### 2.2. p16/Ki67 Immunostaining

**Figure 1:**
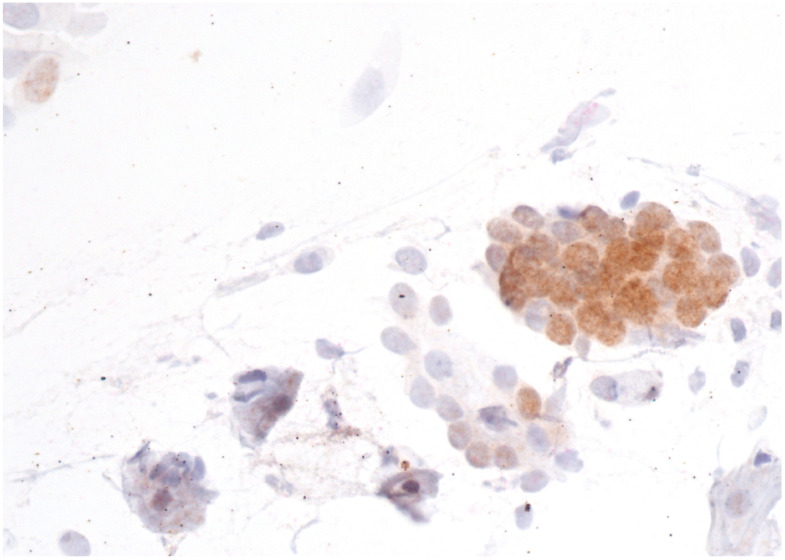
AGC-NOS with negative p16/Ki67 immunostaining. The brown color indicates a positive p16 reaction. However, the Ki67 signal is negative, as there is no red color in the image. Scale bar: 400×.

**Figure 2:**
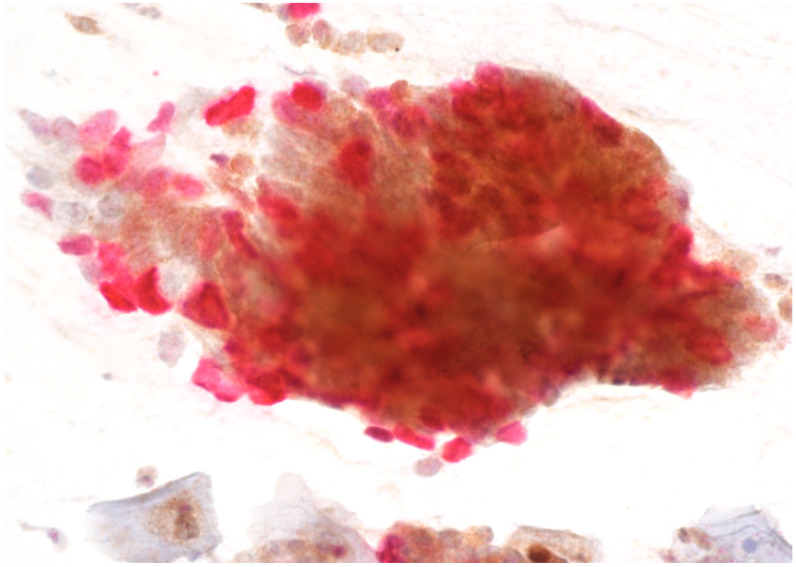
AGC-FN with positive p16/Ki67 immunostaining. Scale bar: 400×.

**Figure 3:**
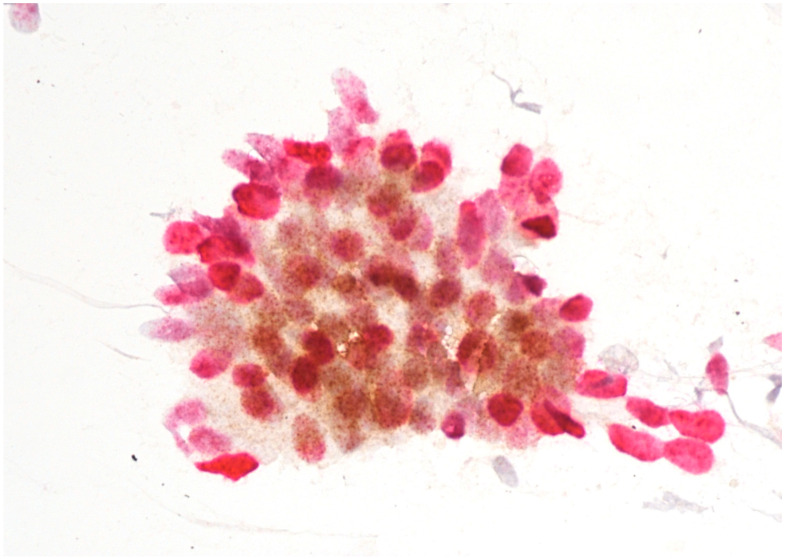
AIS with positive p16/Ki67 immunostaining. Scale bar: 400×.

**Figure 4:**
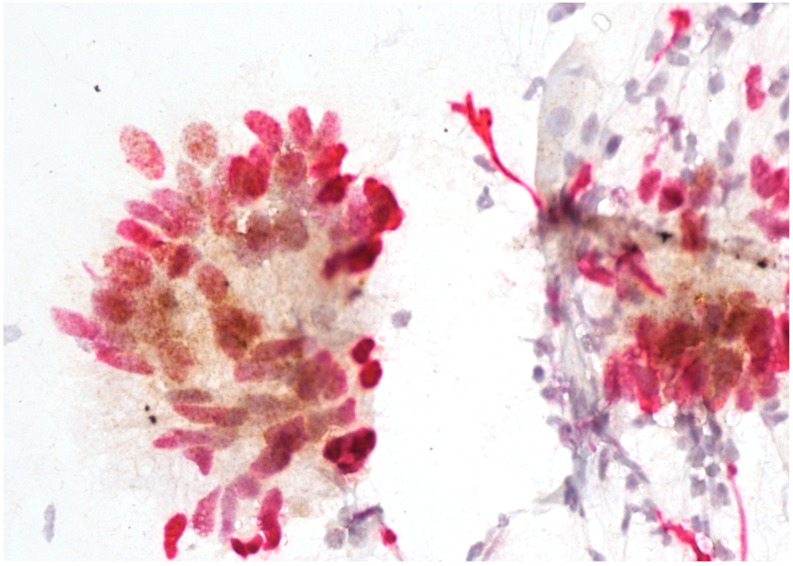
Adenocarcinoma with positive p16/Ki67 immunostaining. Scale bar: 400×.

Immunostaining was performed with p16/Ki67 immunostaining (CINtec PLUS, Ventana Medical Systems, Tucson, AZ, USA), according to the manufacturer’s instructions, retrospectively on all Pap smears, which were interpreted as AGC-NOS, AGC-FN, AIS and A-CA. Decolorization of the Pap smear was performed, and the entire slide was stained with p16 and Ki67. The reaction was regarded as positive when a brown color (which indicated positive p16 signal) and a red color (which indicated positive Ki67 signal) were present. The result was considered positive when most of the clusters of cells (more than 50%) were positive. According to the manufacturer’s instructions, the reaction is positive when at least one cell is stained positive. In glandular abnormalities, atypical cells are usually present in clusters and are not single. Examples of negative and positive immunostaining are shown in Figure 1, Figure 2, Figure 3 and Figure 4. The result of immunohistochemistry was not dependent on morphological criteria. A cytopathologist and a cytotechnologist independently assessed the slides. The final histological diagnosis was not known to the examiners at the time of DS assessment.

### 2.3. Statistical Analysis

Statistical analysis was performed with SPSS software, version 24.0 (IBM, Armonk, NY, USA). Glandular cytological changes were compared to the final histological results. For the purpose of a more transparent presentation of our results, we calculated the diagnostic accuracy of DS for three histological groups: glandular changes (AIS+: adenocarcinoma in situ or adenocarcinoma of the uterine cervix), squamous changes (CIN3+: CIN3 or squamous-cell carcinoma of the uterine cervix) and tumors of the upper reproductive tract: EC/OC (endometrial or ovarian cancer). For each group, we report the positive predictive value (PPV), negative predictive value (NPV), sensitivity (true positive rate), specificity (true negative rate), false positive rate and false negative rate.

## 3. Results

A total of 96 patients with glandular cell abnormalities on cervical cytology were included in the study group. The relative percentages of glandular abnormalities were as follows: AGC-NOS, 65 patients (67.7%); AGC-FN, 20 patients (20.8%); AIS, 5 patients (5.2%); and A-CA, 6 patients (6.3%). With regard to the final histological results, low-grade cervical precancerous lesions or normal histology were present in 59 patients (61.5%), AIS+ in 14 patients (14.6%), CIN3+ in 8 patients (8.3%) and EC/OC in 15 patients (15.6%). Most cases of endometrial cancer were well-differentiated endometrioid adenocarcinomas, and both cases of ovarian cancer were high-grade serous ovarian cancers. The detailed final histological diagnoses obtained from the medical records are presented in Table 1, and final histology results according to the cytology diagnoses are presented in Table 2.

The DS results according to the cytological diagnosis are presented in Table 3.

DS was positive in 13.8% of AGC-NOS smears and in 80% of AIS smears. On the other hand, only 33.3% of the smears diagnosed as A-CA were DS-positive. The differences in DS positivity among different cytology groups were statistically significant (chi-square = 18.794; *p* ˂ 0.05).

The DS results according to the final histology results are presented in Table 4.

DS was positive in 8.5% of patients with normal results or low-grade cervical lesions at final histology and in 85.7% of patients with AIS+ at final histology. The differences among all histology groups were statistically significant (chi-square = 41.142; *p* ˂ 0.05).

The diagnostic accuracy of DS is presented in Table 5.

The specificity of DS for the detection of CIN3+ was 78.2% in the whole population and 91.5% in the AGC-NOS group, respectively. The rate of false positive results in the AGC-NOS subgroup was 8.5%. The specificities of DS for the detection of AIS+ and EC/OC were 84.1% and 72.8%, respectively, for the whole study population and 88.7% and 86.4%, respectively, for the AGC-NOS subgroup.

## 4. Discussion

In recent years, the relative incidence of cervical adenocarcinoma among all cervical cancers has increased, and the incidence of squamous cancer has decreased [19,20]. In women aged 20–49, the relative percentages of cervical adenocarcinoma have increased by two to three times from 10–15% in the past to 20% in the present [19,21]. In addition, the incidence of AIS has increased in the group of women aged 25–39 [19]. In consequence, glandular cytological changes on cervical smears have gained increasing attention in recent years [22].

From these data, it seems that cervical cancer screening programs have not been effective in decreasing the incidence of cervical adenocarcinoma [19,23]. The reason probably lies in inconclusive cervical cytology for the detection of cervical adenocarcinoma [17]. In addition, the rate of HPV negativity among cervical adenocarcinomas is 15–20%, and these lesions are expected to be less affected by HPV screening [24].

The rate of glandular pathology detected on follow-up after atypical glandular cells on cervical smears varies among published studies. An American research group reported that AIS or cervical adenocarcinoma was detected in only 1.9% of cases after a one-year follow-up of 3007 patients with cervical cancer smears with glandular abnormalities [25]. Others reported the relative incidence of cervical adenocarcinoma following atypical glandular cells on cervical cytology among patients with available follow-up to range between 5% and 33% [26,27,28].

p16/Ki67 DS has been proposed as a valuable addition in the screening of cervical glandular lesions as an objective test that could decrease interobserver variability in diagnosing atypical glandular cells [17]. In our study, 85.7% of patients with a subsequent diagnosis of AIS+ had positive DS. By contrast, 91.5% of patients with a negative follow-up or a low-grade lesion on follow-up had negative DS. In a recent Japanese study on 142 patients with benign, atypical and malignant glandular cells of the cervix, DS was positive in all patients, with a subsequent diagnosis of cervical adenocarcinoma [17]. In addition, there were no negative cases among patients with a subsequent diagnosis of AIS [17]. On the other hand, a Chinese research group reported 71.4% DS positivity in patients with a subsequent diagnosis of cervical adenocarcinoma, but there were only seven cases [29].

There is only a limited number of studies published on the utility of DS on glandular cell abnormalities [30,31]. An American retrospective analysis reported on 122 cervical cytology specimens, which included 18 cases with glandular cell abnormalities [31]. In total, 55.6% of all cervical smears containing atypical glandular cells were DS-positive. This study found six patients with AIS+, and all were DS-positive [31]. Italian researchers evaluated the diagnostic performance of DS for the detection of AIS and AIS with early invasion on a group of 63 liquid-based cytology cases [30]. There were 40 patients with a subsequent diagnosis of AIS or AIS with early invasion and 16 patients with negative follow-up. Among the patients with negative follow-up, only one was DS-positive, and among those with positive follow-up, 93.8% were DS-positive. The sensitivity of DS for the detection of AIS with or without early invasion was 97.4%, and the specificity was 83.3% [30].

The reported sensitivity of cervical cytology for the detection of cervical adenocarcinoma is 45% and 75% after the exclusion of inadequate samples [32]. Our results show higher sensitivity rates of DS for the detection of AIS+ for AGC-NOS and AGC-FN subgroups. Among 65 patients with AGC-NOS in our study, 86.2% were DS-negative. The previously mentioned Japanese study reported 53.3% (8 out of 15) DS-negative results in patients with AGC-NOS [17]. The specificity of DS for the detection of AIS+ in the AGC-NOS cytology subgroup in our study was 88.7%, and the sensitivity was 66.7%. In the study by Ryu et al., positive DS was associated with a high possibility of cervical adenocarcinoma, and this association was particularly strong in patients younger than 39 years. On the other hand, they reported a low probability of cervical adenocarcinoma in patients younger than 60 years old with negative DS [17]. On a large sample of 1630 patients with AGC-NOS cytology, Pradhan et al. reported AIS and cervical adenocarcinoma at follow-up in 22 patients (1.5%) [25]. Contrary to AGC-NOS, where squamous glandular lesions are the most frequent finding on follow-up, AGC-FN is more commonly associated with glandular lesions of the cervix and the endometrium [22]. In a large single-center analysis of over 3000 smears with AGC, there were only 26 cases of AGC-FN, and 8 of these (30.8%) were AIS+ on follow-up [25]. Our results show a similar pattern, with CIN3+ being more common compared to AIS+ in the AGC-NOS group and vice versa in the AGC-FN group. It has been postulated that DS might be a useful adjunctive tool for cervical adenocarcinoma screening when atypical glandular cells are difficult to identify. However, especially in patients over 40 years old, DS is not able to exclude glandular lesions that do not originate in the cervix [17].

DS was positive in 65% of patients with squamous intraepithelial lesions and squamous-cell cancer of the cervix in our study. The specificity of DS for the detection of CIN3+ in the AGC-NOS population was 91.5%, and a negative result was associated with a low probability of CIN3+. Ryu et al. reported on four patients with CIN2+, all of whom had positive DS [17]. According to the literature, the most common cervical lesion following AGC-NOS cytology is squamous intraepithelial lesion [22], and therefore, DS might provide additional information in cervical cancer screening.

In our study, there were fifteen patients with EC/OC, among which only three had positive DS. This might be due to the fact that most endometrial carcinomas in our study were of endometrioid histology. This histological entity usually shows focal p16 positivity, and the cases with focal DS positivity were regarded as negative in our study [33]. On the other hand, the majority of cases of serous uterine carcinoma were reported to have diffusely positive DS [17].

To summarize, our results indicate that DS could be a useful addition in the screening of glandular abnormalities of the uterine cervix. A large majority of patients with a subsequent diagnosis of AIS or cervical adenocarcinoma had positive immunostaining (Figure 2, Figure 3 and Figure 4), whereas the majority of those with low-grade lesions or normal histology had negative immunostaining (Figure 1). Taking into consideration the difficulties in diagnosing cervical glandular lesions, this method might be a useful addition to the diagnostic process.

The major strengths of our study were the available follow-up data and surgical specimens for all participants, which enabled direct comparisons for the whole study group. Histologic evaluation after AGC in cervical cytology is not uniform, and therefore, a significant variation in histologic outcome exists [22]. In addition, glandular and squamous malignant and premalignant lesions are more frequently detected in surgical specimens compared to the cases where only biopsies are available [22].

Our study has several limitations. The sample is relatively small but comparable to previously published studies [17]. Taking into consideration all the limitations that exist with the diagnosis of glandular cervical lesions, the real incidences of glandular lesions of the uterine cervix can only be adequately assessed in large-scale studies [22]. In addition, we did not divide our DS-positive cases into diffusely and focally positive categories, as Ryu et al. did [17]. However, in their study, it was suggested that only diffusely positive cases should be considered positive since a significant percentage of focally positive cases exhibited a heterogenous staining pattern [17]. Another limitation of our study was the low rate of cervical adenocarcinomas, which prohibited us from calculating the diagnostic performance of DS for AIS and cervical adenocarcinoma according to different cytology results.

## 5. Conclusions

In conclusion, our study confirms high specificity and sensitivity values of the p16/Ki67 DS of glandular cell abnormalities for the detection of AIS+. Further research on larger samples is needed in order to confirm these results.

## Figures and Tables

**Table 1:** Final histological results from the study group (N = 96).

Final Histology	No. of Cases (%)
Negative histology	54 (56.3%)
CIN1	5 (5.2%)
CIN3	5 (5.2%)
AIS	10 (10.4%)
Squamous cervical cancer	3 (3.1%)
Cervical adenocarcinoma	4 (4.2%)
Endometrial cancer	13 (13.5%)
HGSOC	2 (2.1%)

**Table 2:** Follow-up histology obtained from surgical specimens according to different categories of atypical glandular cells (AGC; Bethesda classification). The results are presented as No. of patients (%); N = 96.

	Negative/CIN1	CIN3+	AIS+	EC/OC	Total
AGC-NOS	50 (76.9)	6 (9.2)	3 (4.6)	6 (9.2)	65
AGC-FN	8 (40.0)	2 (10.0)	6 (30.0)	4 (20.0)	20
AIS	0	0	5 (100.0)	0	5
A-CA	1 (16.7)	0	0	5 (83.3)	6
Total	59	8	14	15	96

AGC-NOS: atypical glandular cells, not otherwise specified; AGC-FN: atypical glandular cells, favor neoplastic; AIS: adenocarcinoma in situ; A-CA: adenocarcinoma; CIN1: cervical intraepithelial neoplasia grade 1; CIN3+: cervical intraepithelial neoplasia grade 3 or higher; AIS+: adenocarcinoma in situ or cervical adenocarcinoma; EC/OC: endometrial carcinoma/ovarian carcinoma.

**Table 3:** DS results according to different cytology results.

Cervical Cytology	DS-Negative (%)	DS-Positive (%)	Total
AGC-NOS	56 (86.2)	9 (13.8)	65
AGC-FN	10 (50.0)	10 (50.0)	20
AIS	1 (20.0)	4 (80.0)	5
A-CA	4 (66.7)	2 (33.3)	6
Total	71 (74.0)	25 (26.0)	96

DS: p16/Ki67 dual staining; AGC-NOS: atypical glandular cells, not otherwise specified; AGC-FN: atypical glandular cells, favor neoplastic; AIS: adenocarcinoma in situ; A-CA: adenocarcinoma.

**Table 4:** DS results according to final histology results.

Final Histology	DS-Negative (%)	DS-Positive (%)	Total
Negative/CIN1	54 (91.5)	5 (8.5)	59
CIN3+	3 (37.5)	5 (62.5)	8
AIS+	2 (14.3)	12 (85.7)	14
EC/OC	12 (80.0)	3 (20.0)	15
Total	71 (74.0)	25 (26.0)	96

DS: p16/Ki-67 dual staining; CIN1: cervical intraepithelial neoplasia grade 1; CIN3+: cervical intraepithelial neoplasia grade 3 or squamous-cell cervical carcinoma; AIS+: adenocarcinoma in situ or cervical adenocarcinoma; EC/OC: endometrial cancer or ovarian cancer.

**Table 5:** Diagnostic performance of DS for the detection of CIN3+, AIS+ and EC/OC. All values are in %.

	Sensitivity	Specificity	PPV	NPV	FPR	FNR
CIN3+						
Total	62.5	78.2	20.8	95.8	21.8	37.5
AGC-NOS	66.7	91.5	44.4	96.4	8.5	33.3
AGC-FN	57.1	50.0	30.8	75.0	50.0	42.9
AIS	NA	20.0	0	100.0	80.0	NA
A-CA	NA	66.7	0	100.0	33.3	NA
AIS+						
Total	87.5	84.1	48.0	97.2	15.9	14.3
AGC-NOS	66.7	88.7	22.2	98.2	11.3	33.3
AGC-FN	100.0	71.4	60.0	100.0	28.6	0.0
AIS	80.0	NA	100.0	0.0	NA	20.0
A-CA	NA	66.7	0.0	100.0	33.3	NA
EC/OC						
Total	20.0	72.8	12.0	83.0	27.2	20.0
AGC-NOS	16.7	86.4	11.1	91.1	13.6	83.3
AGC-FN	0.0	37.5	0.0	60.0	62.5	100.0
AIS	NA	20.0	0.0	100.0	80.0	NA
A-CA	0.0	100.0	100.0	25.0	0.0	100.0

DS: p16/Ki-67 dual staining; AGC-NOS: atypical glandular cells, not otherwise specified; AGC-FN: atypical glandular cells, favor neoplastic; AIS: adenocarcinoma in situ; A-CA: adenocarcinoma; CIN3+: cervical intraepithelial neoplasia grade 3 or higher; AIS+: adenocarcinoma in situ or cervical adenocarcinoma; EC/OC: endometrial carcinoma/ovarian carcinoma; PPV: positive predictive value; NPV: negative predictive value; FPR: false positive rate; FNR: false negative rate.

## Data Availability

Data are contained within the article.
